# Comparative analysis of a sex chromosome from the blackchin tilapia, *Sarotherodon melanotheron*

**DOI:** 10.1186/s12864-016-3163-7

**Published:** 2016-10-18

**Authors:** William J. Gammerdinger, Matthew A. Conte, Jean-François Baroiller, Helena D’Cotta, Thomas D. Kocher

**Affiliations:** 1 Department of Biology, University of Maryland, College Park, MD 20742 USA; 2 UMR-ISEM, CIRAD, Montpellier, France

**Keywords:** African cichlids, Sex chromosome evolution, Sex-determination

## Abstract

**Background:**

Inversions and other structural polymorphisms often reduce the rate of recombination between sex chromosomes, making it impossible to fine map sex-determination loci using traditional genetic mapping techniques. Here we compare distantly related species of tilapia that each segregate an XY system of sex-determination on linkage group 1. We use whole genome sequencing to identify shared sex-patterned polymorphisms, which are candidates for the ancestral sex-determination mutation.

**Results:**

We found that *Sarotherodon melanotheron* segregates an XY system on LG1 in the same region identified in *Oreochromis niloticus*. Both species have higher densities of sex-patterned SNPs, as well as elevated number of ancestral copy number variants in this region when compared to the rest of the genome, but the pattern of differentiation along LG1 differs between species. The number of sex-patterned SNPs shared by the two species is small, but larger than expected by chance, suggesting that a novel Y-chromosome arose just before the divergence of the two species. We identified a shared sex-patterned SNP that alters a *Gata4* binding site near *Wilms tumor protein* that might be responsible for sex-determination.

**Conclusions:**

Shared sex-patterned SNPs, insertions and deletions suggest an ancestral sex-determination system that is common to both *S. melanotheron* and *O. niloticus*. Functional analyses are needed to evaluate shared SNPs near candidate genes that might play a role in sex-determination of these species. Interspecific variation in the sex chromosomes of tilapia species provides an excellent model system for understanding the evolution of vertebrate sex chromosomes.

**Electronic supplementary material:**

The online version of this article (doi:10.1186/s12864-016-3163-7) contains supplementary material, which is available to authorized users.

## Background

Natural selection alters the local genomic environment around newly evolved sex-determination loci in two ways. First, selection favors the accumulation of sexually antagonistic alleles and their association with the appropriate X- or Y-haplotype. Second, selection also favors a reduction in the rate of recombination between the proto-X and proto-Y to preserve the associations between the sexually antagonistic alleles and the sex-determination locus [1, 2]. The process is cyclic, in that selection will favor the recruitment of additional sexually antagonistic loci within a growing region of reduced recombination [3]. Once a region of reduced recombination is established, deleterious mutations will begin to accumulate via Muller’s Ratchet [4]. Through these mechanisms, the region of differentiation between the X- and Y-chromosome grows, creating a series of evolutionary strata from the oldest and most decayed regions to the newest and least decayed [5]. Structural rearrangements, such as inversions, are one mechanism that reduces recombination and contributes to the creation of evolutionary strata on sex chromosomes [1, 2, 5].

The initial sex-determination loci are buried within the oldest evolutionary strata because the first selectively favored chromosomal rearrangement encompasses both the sex-determination locus and at least one sexually antagonistic locus. Traditional genetic mapping techniques cannot be used to fine map the sex-determination locus because of the lack of recombination inside these structural polymorphisms. An alternative approach is to identify shared ancestral polymorphisms inside the deepest and most decayed stratum among species that share a common ancestral sex-determination mechanism.

Cichlid fishes are a model system for studying evolutionary processes [6]. Sex-determination has evolved rapidly among African cichlids. Sex-determination loci have been identified on linkage groups (LG) 1 (XY), 3 (WZ) and 23 (XY) among species of tilapia, and on linkage groups 5 (WZ), 7 (XY) and possibly others in haplochromine cichlids [7–9]. The sex determination locus on LG23 appears to be a duplication of *amh*, but the sex-determination genes on the other chromosomes have not yet been identified [10, 11].

Previous studies have mapped the sex-determination locus on linkage group 1 in the Nile tilapia (*Oreochromis niloticus*) to a region of a few centimorgans [8, 12–14]. We recently identified a high density of sex-patterned SNPs on LG1 in the region from 10.1 to 18.9 Mb [15]. This 8.8 Mb region is significantly enriched for missense mutations and likely corresponds to an inversion that restricts recombination between the X- and Y-chromosomes.

The blackchin tilapia, *Sarotherodon melanotheron*, is a sister group to the genus *Oreochromis* [16] and has received relatively little attention with respect to sex-determination. One study reported that the chromosomes of *S. melanotheron* were homomorphic with a chromosome count consistent with that of other species of *Oreochromis* [17]. In this study we identify the sex-determination region in *S. melanotheron*, characterize the shared regions of differentiation on LG1 between *O. niloticus* and *S. melanotheron* and catalog shared SNPs that may be responsible for sex-determination in these species.

## Methods

### Materials

*Sarotherodon melanotheron* were originally collected from Lake Guiers, Senegal. The individuals sampled are the 4^th^ generation progeny of the wild-caught fish. Fins were sampled from a total of 22 male and 22 female fish and preserved in a salt-DMSO preservation solution. The sex ratio of the family was 50:50.

### Sequencing

DNA was extracted from each fin-clip by phenol-chloroform extraction using phase-lock gel tubes (5Prime, Gaithersburg, Maryland). DNA concentrations were measured by fluorescence spectrometry and normalized during library preparation. DNA from 21 males and 22 females were then separately pooled (one likely male was excluded due to ambiguous gonads). Sequencing libraries were constructed using the TruSeq DNA PCR-Free LT Kit (Illumina, San Diego, California). A full lane of Illumina 183 bp paired-end sequencing was performed for each sex.

For comparative analyses, we reanalyzed whole genome sequencing data for male and female *Oreochromis niloticus* from our previous study [15]. These data consist of 100 bp paired-end reads from separate male and female DNA pools.

### Read mapping

Reads that did not pass Illumina CASAVA 1.8 filtering were removed. Read qualities for both lanes were analyzed using FastQC [18]. The reads were then mapped to the *O. niloticus* reference assembly with Bowtie2 using the --*very-sensitive* setting and a minimum insert length of 200 bp [19, 20]. Variants were called using GATK in the *UnifiedGenotyper* mode [21]. Read qualities with a PHRED score of at least 20 were required for variant calling.

### Identification of sex-patterned SNPs

A custom script was used to identify sites showing a sex-patterned signature. Sex_SNP_Finder_GA.pl is an expansion of the Sex_SNP_finder_now.pl script released previously [15, 22]. Sex-patterned SNPs are sites that are fixed or nearly fixed in the homogametic sex and in a frequency between 0.3 and 0.7 in the heterogametic sex. We required a minimum read depth of ten and a minimum allele count of two. We assessed the overall density of sex-patterned SNPs in 10 kb non-overlapping windows. We also counted regions enriched for sex-patterned SNPs by identifying non-overlapping 10 kb windows containing at least 10 sex-patterned SNPs.

In addition to finding sex-patterned SNPs, the updated script calculates F_ST_, d_*xy*_, d_*a*_, Nei’s D and C_*P*_ for each nucleotide position across the genome. F_ST_ is calculated in accordance with the method used in PoPoolation2, except that we calculated values at each site instead of by windows [23]. Due to numeric difficulties handling the upper bound of Nei’s D, the calculation is modified for alternatively fixed positions to make the calculation based upon the maximum coverage level specified by the user. C_*P*_ is a metric of population differentiation that ranges from 0 (no population differentiation) to 1 (complete population differentiation), where *x*_*i*_ and *y*_*i*_ are the frequencies of each allele in populations *x* and *y* [24].

$$ {C}_p = {\displaystyle \sum_{i=1}^n}\frac{1}{2}\left|\ {x}_i - {y}_i\ \right| $$

### Functional annotation

The functional significance of sex-patterned SNPs was evaluated with SnpEff and SnpSift using the gene annotations obtained from NCBI (RefSeq release 70) [25, 26]. We excluded mRNA models that did not have full-length protein coding sequences. Non-synonymous substitutions were subsequently evaluated with PROVEAN to predict functional impacts on protein structure [27]. Three missense mutations, R203C in XM_003448054.2 (LG6), E1235V in XM_003438386.2 (LG13) and G215A in XM_005466093.1 (LG22), were excluded from the PROVEAN analysis due to low quality gene annotations as defined by PROVEAN. Missense mutations harboring PROVEAN scores less than the recommended threshold of −2.5 were considered deleterious.

### Identification of shared sex-specific SNPs

Sex-patterned SNPs in *S. melanotheron* were compared to the sex-patterned SNPs identified in *O. niloticus*, which also segregates a sex-determination system on LG1. The null hypothesis for the expected number of shared sex-patterned SNPs was calculated by multiplying the frequency of sex-patterned SNPs within the previously identified region from 10.1 to 18.9 Mb on LG1 in *O. niloticus*, by the frequency of sex-patterned SNPs within the same region in *S. melanotheron* by the size of the region.

### Transcription factor binding site analysis

SNPs that were sex-patterned and shared between *O. niloticus* and *S. melanotheron* were inspected to ensure that they shared common X- and Y-alleles. Flanking regions for the SNPs passing this criteria were extracted using Bedtools and compared to the JASPAR CORE Vertebrata 2016 database to identify possible transcription factor binding sites [28, 29]. A JASPAR relative score threshold of 0.80 was used to assess the significance of putative binding sites.

### Identification of copy number variants

Copy number variation within the *S. melanotheron* and *O. niloticus* datasets was assessed with VarScan 2, using a minimum window size of 100 bp, a maximum window size of 1 kb and *amp* and *del* thresholds equal to 0.2 [30]. A custom script (Varscan_multiple.pl) was developed to find conserved copy number variants between *n*-number of VarScan 2 comparisions [31]. We then utilized a non-overlapping window approach, which excluded assembly gaps from the windows, to quantify the density of these conserved copy number variants within 10 kb windows across the genome.

### Statistical assessment

Mann–Whitney U-tests were used to determine if a region was enriched for sex-patterned SNPs or conserved copy number variants. All testing used 10 kb non-overlapping windows as samples. A Bonferroni correction was applied to control for 12 comparisons resulting in a significance threshold of *α*=0.004167.

## Results

### Sequencing and mapping of reads

We obtained a total of ~160 million and ~162 million 183 bp paired-end reads from the female and male pools of *S. melanotheron*, respectively. The alignment rates to the *O. niloticus* reference sequence were 85.50 and 85.69 %, respectively. The mean coverage in the female pool was 35.12 reads, while the mean coverage in the male pool was 35.75 reads.

We obtained a total of ~219 million and ~202 million 100 bp paired-end reads from the female and male pools of *O. niloticus*, respectively. The alignment rates to the *O. niloticus* reference were 90.49 and 89.96 %, respectively. The mean coverage in the female pool was 27.17 reads, while coverage in the male pool was 26.84 reads.

### F_ST_ differentiation in *S. melanotheron*

**Fig. 1:**
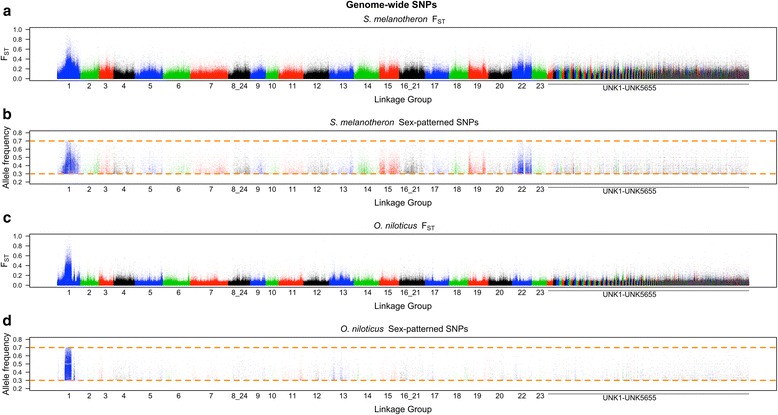
Whole genome survey of **a** F_ST_ in *S. melanotheron*, **b** sex-patterned SNPs in *S. melanotheron*, **c** F_ST_ in *O. niloticus* and **d** sex-patterned SNPs in *O. niloticus*

**Fig. 2:**
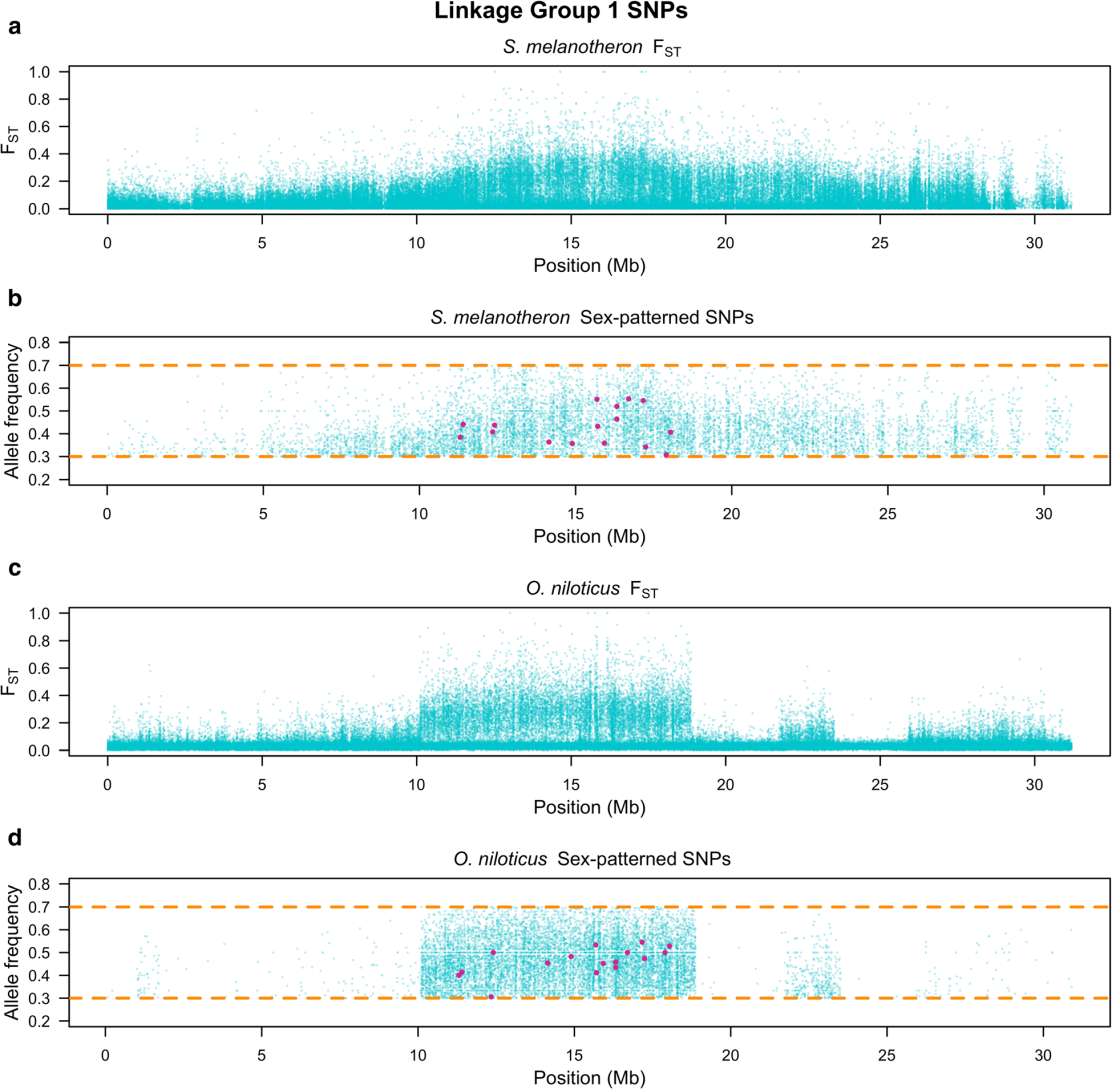
Linkage group 1 survey of **a** F_ST_ in *S. melanotheron*, **b** sex-patterned SNPs in *S. melanotheron*, **c** F_ST_ in *O. niloticus* and **d** sex-patterned SNPs in *O. niloticus*. The *red points* represent the shared sex-patterned SNPs between *O. niloticus* and *S. melanotheron*

Examination of the whole genome F_ST_ plot comparing male and female *S. melanotheron* identifies a strong signal on LG1 (Fig. 1). A closer examination of LG1 reveals that this region overlaps with the previously identified XY sex-determination region in *O. niloticus* (Fig. 2). The region of divergence on LG1 is broader in *S. melanotheron* than in *O. niloticus* and spans from approximately 10.1 to 28 Mb. The boundaries of the differentiated region in *S. melanotheron* are gradual, unlike the sharp boundaries found in *O. niloticus* on LG1 from 10.1 to 18.9 Mb and from 21.7 to 23.6 Mb.

**Fig. 3:**
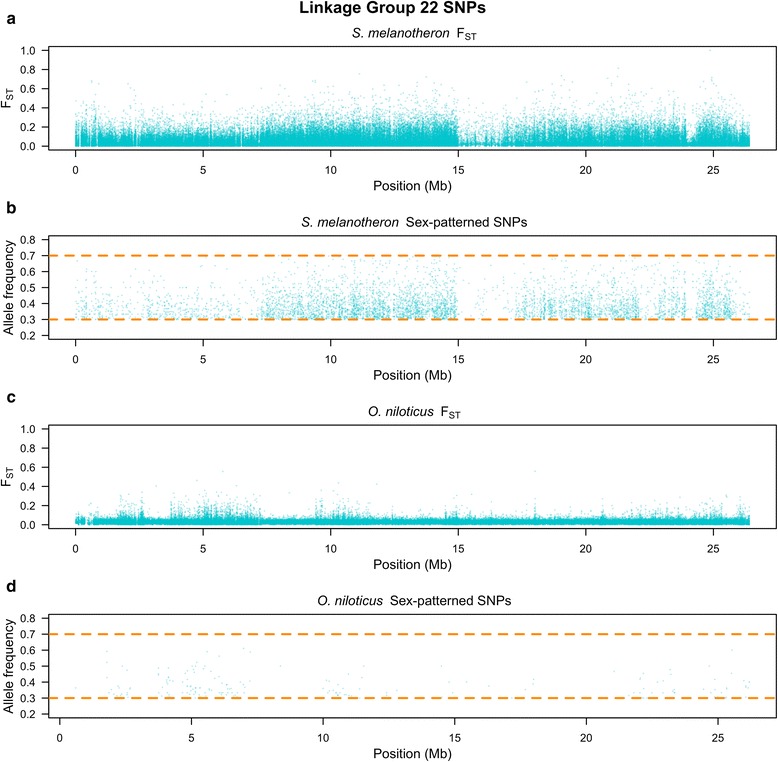
Linkage group 22 survey of **a** F_ST_ in *S. melanotheron*, **b** sex-patterned SNPs in *S. melanotheron*, **c** F_ST_ in *O. niloticus* and **d** sex-patterned SNPs in *O. niloticus*

*S. melanotheron* also shows indications of divergence between the sexes on LG22. There is no previous evidence to suggest that this region is associated with sex in any cichlid species and the signal is not as strong as on LG1 (Fig. 3 and Table 1). Importantly, *S. melanotheron* shows no strong sex-patterned signal from LG3, a region known to harbor a sex-determination locus in *Pelmatotilapia mariae* and some members of *Oreochromis* [8].

**Table 1:** Average F_ST_ across all polymorphic sites along with total counts and densities of sex-patterned SNPs across the regions of differentiation in *S. melanotheron* and *O. niloticus*

	Average F_ST_ across polymorphic sites	Sex-patterned SNPs (per Mb)	Enriched 10 kb windows (per Mb)	Missense SNPs (per Mb)	PROVEAN SNPs < −2.5 (per Mb)	Synonymous SNPs (per Mb)
*S. melanotheron*						
LG1: 10.1 Mb-18.9 Mb	0.111	5342 (607.05)	193 (21.93)	61 (6.93)	6 (0.68)	121 (13.75)
LG1: 18.9 Mb-28 Mb	0.081	2702 (296.92)	52 (5.71)	38 (4.18)	5 (0.55)	40 (4.40)
LG22	0.057	5709 (216.16)	80 (3.03)	42 (1.59)	9 (0.34)	108 (4.09)
Rest of the genome	0.034	36,335 (41.13)	123 (0.14)	669 (0.76)	103 (0.12)	962 (1.09)
Total		50,088	448	810	123	1231
*O. niloticus*						
LG1: 10.1 Mb-18.9 Mb	0.039	10,792 (1226.36)	517 (58.75)	159 (18.07)	25 (2.84)	256 (29.09)
LG1: 21.7 Mb-23.6 Mb	0.024	399 (210.00)	3 (0.33)	5 (2.63)	1 (0.53)	6 (3.16)
Rest of the genome	0.021	8602 (9.38)	19 (0.02)	167 (0.18)	27 (0.03)	180 (0.20)
Total		19,793	539	331	53	442

### Sex-patterned SNPs

We found a total of 50,088 SNPs in *S. melanotheron* and 19,793 SNPs in *O. niloticus* fitting the sex-patterned criteria. There were 448 and 539 non-overlapping 10 kb windows with at least 10 sex-patterned SNPs in *S. melanotheron* and *O. niloticus*, respectively (Table 1). The highest densities of sex-patterned SNPs occurred between 10.1 and 18.9 Mb in both *O. niloticus* and *S. melanotheron*. Sex-patterned SNPs found on LG1 between 10.1 and 18.9 Mb are at significantly higher densities in this region than any other region in either species (*p* < 0.0001, for all five comparisons to other regions noted in the table).

In *S. melanotheron*, the region on LG1 from 18.9 to 28 Mb, as well as LG22, both showed a significantly higher density of sex-patterned SNPs when compared to the rest of the genome (*p* < 0.0001, for both comparisons). The region on LG1 from 18.9 to 28 Mb also demonstrated a significantly higher density of sex-patterned SNPs than LG22 (*p* < 0.0001). In *O. niloticus*, the region on LG1 from 21.7 to 23.6 Mb showed a significantly higher density of sex-patterned SNPs than the rest of the genome (*p* < 0.0001).

### Functional impacts

We evaluated the functional impacts of the sex-patterned SNPs and found a total of 810 missense and 1231 synonymous mutations in *S. melanotheron*. Table 1 shows the average F_ST_ across all polymorphic SNPs within each region as well as the genomic distribution of each subset of the sex-patterned SNPs: total sex-patterned SNPs, 10 kb windows enriched for sex-patterned SNPs, missense SNPs, missense SNPs with a PROVEAN score less than −2.5 and synonymous mutations. The density of each class of sex-patterned SNPs in *S. melanotheron* was consistently from highest to lowest: LG1 between 10.1 and 18.9 Mb, LG1 between 18.9 and 28 Mb, LG22, then across the rest of the genome. There were seven stop codon gains or losses, but only one mapped to a region of divergence on LG1 (Additional file 1).

In *O. niloticus* we identified a total of 331 missense and 442 synonymous mutations. The density of each class of sex-patterned SNPs in *O. niloticus* was consistently from highest to lowest: LG1 between 10.1 and 18.9 Mb, LG1 between 21.7 and 23.6 Mb, then across the rest of the genome. There were six stop codon gains or losses, but none mapped to the regions of divergence (Additional file 2). These counts differ slightly from Gammerdinger et al., 2014 due to a difference in the gene annotation versions used. There were no conserved stop codon gains or losses between *S. melanotheron* and *O. niloticus*.

### Copy number variants

We identified a significantly higher density of conserved duplications and deletions between *O. niloticus* and *S. melanotheron* on both LG1 from 10.1 to 18.9 Mb (*p* < 0.0001) and LG1 from 21.7 to 23.6 Mb (*p* < 0.0002) when each is compared to the rest of the genome. However, we did not detect any significant difference in the density of duplications and deletions conserved between the species in these divergent regions on LG1.

### Shared SNPs

There are 42 sex-patterned SNPs conserved between *S. melanotheron* and *O. niloticus*, compared to the null expectation of 6.55 conserved SNPs. We examined all 42 positions to confirm that the X- and Y-alleles were consistent in the two species. There were 13 SNPs where the X- and Y-alleles are switched between species (e.g. when the X-allele in *O. niloticus* is the Y-allele in *S. melanotheron*). Eight sites showed a pattern where the Y-alleles are different but the X-alleles were the same in each lineage. This narrowed the list to 21 SNPs where the X- and Y-alleles were shared between the two species. We filtered these 21 SNPs by genomic position. Sixteen fell within the region on LG1 between 10.1 and 18.9 Mb, one lay on LG14 and four fell on unanchored scaffolds (Additional files 3 and 4). Of these shared SNPs, none were missense mutations and only one was a synonymous mutation. The rest were located in non-coding regions. We identified shared sex-patterned SNPs within 20 kb of two previously identified candidate genes for sex-determination on LG1 [15]. One was 19 kb downstream of *Wilms’ tumor protein* and the other was 5 kb downstream of *Ras association domain containing protein 10*.

We used JASPAR to examine a short region flanking each of the 21 shared sex-patterned SNPs for transcription factor binding sites (Additional file 5). A sex-patterned SNP at position 14,895,959 on LG1, near *Wilms’ tumor protein*, resulted in a loss of a *Gata4* binding site. The sex-patterned SNP at 11,400,015 on LG1, near *Ras association domain containing protein 10* did not alter binding sites for any transcription factor binding sites known to be involved in the sex-determination network (Additional file 5).

## Discussion

### Evidence for a shared LG1 sex-determination mechanism between 10.1 Mb and 18.9 Mb

The initial line of evidence that *S. melanotheron* shares an ancestral sex-determination mechanism with *O. niloticus* is the overlap of the region harboring sex-determination on LG1 (Figs. 1 and 2). Convergence for sex-determination within the same 8.8 Mb region within a 927 Mb genome, while possible, seems unlikely. This region also contains the highest density of sex-patterned SNPs across the genome in both species. The high density of sex-patterned SNPs explains the elevated sex-related F_ST_ witnessed within this region. Furthermore, the copy number variant analysis demonstrates that the region on LG1 from 10.1 to 18.9 Mb is not significantly more divergent than the region from 21.7 to 23.6 Mb, but it does have significantly more conserved insertions and deletions compared to the rest of the genome. Lastly, the number of shared sex-patterned SNPs is several times more than expected by chance (6.55 shared sex-patterned SNPs). Collectively, the evidence strongly suggests the idea that *O. niloticus* and *S. melanotheron* share a common ancestral sex-determination mechanism on LG1.

### Evidence for a shared sexually antagonistic locus on LG1 between 21.7 Mb and 23.6 Mb

The region from 21.7 to 23.6 Mb on LG1 has a moderately strong signal of differentiation between males and females in both lineages. The region from 18.9 to 28 Mb in *S. melanotheron* includes this region and is the second most densely sex-patterned SNP region in the genome. The region from 21.7 to 23.6 Mb does not have significantly more conserved insertions and deletions than the region from 10.1 to 18.9 Mb, but it does have significantly more conserved insertions and deletions when compared to the rest of the genome. This suggests that the block of differentiation between 21.7 and 23.6 Mb has a shared deep history with the sex-determination region.

Within this region, we found no shared SNPs with a consistent allelic pattern between *O. niloticus* and *S. melanotheron*, but there was a sex-patterned locus in both species where the X- and Y-alleles were switched. This suggests that there could have been an ancestral polymorphism that was alternatively sorted during speciation and the emergence of the sex chromosome. The evidence suggests that the diverged region on LG1 from 21.7 to 23.6 Mb was present during the initial divergence of sex-determination ancestral to both *O. niloticus* and *S. melanotheron* on LG1 from 10.1 to 18.9 Mb.

This linked, differentiated region from 21.7 to 23.6 Mb may harbor a sexually antagonistic locus. However, the sequence between these regions of divergence, from 18.9 to 21.7 Mb, shows differentiation in *S. melanotheron*, but not in *O. niloticus*. Previous theoretical work has suggested that sexually antagonistic loci can be in linkage disequilibrium with the sex-determination locus over large distances while not showing strong signs of differentiation between the two loci [32]. It appears that *O. niloticus* has potentially maintained linkage disequilibrium between these two pockets of differentiation, while *S. melanotheron* has accumulated mutations across this entire region. This theoretical prediction also postulates that the sexually antagonistic region should have a lower level of divergence than the sex-determination region. This proposition is consistent with the data collected in *O. niloticus*.

**Fig. 4:**
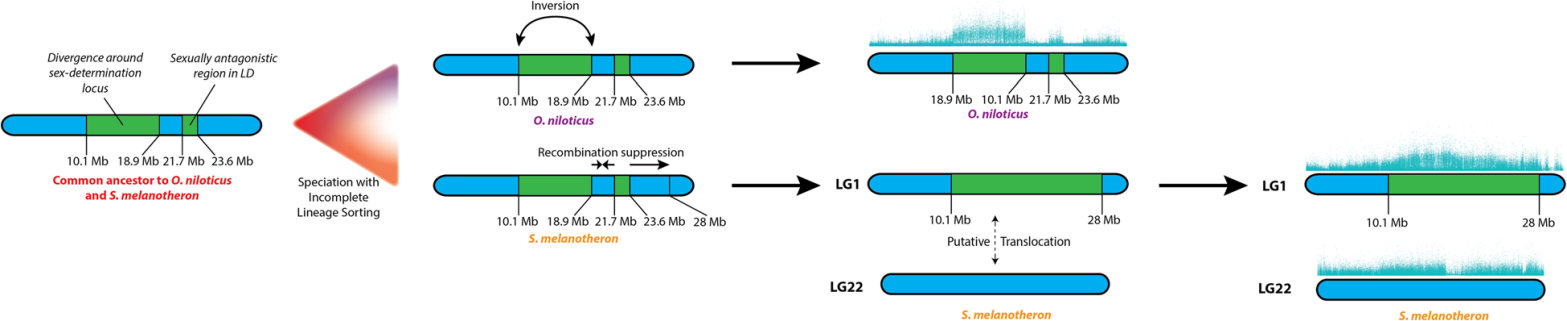
Proposed model for the evolution of the linkage group 1 sex-determination system in tilapia

From our data, it is unclear if the intervening region from 18.9 to 21.7 Mb diverged initially and then recombination was restored in the *O. niloticus* lineage or if these were initially two separate regions of divergence held together by sexually antagonistic selection and recombination was subsequently reduced across the entire region in the *S. melanotheron* lineage. Figure 4 illustrates the latter sequence of events, but the former sequence of events is also plausible. Sequences of more species harboring the LG1 sex-determination system might distinguish these two hypotheses.

Alternatively, this region could represent a misassembly in the *O. niloticus* reference genome or a Y-specific structural rearrangement.

### Evidence for speciation soon after sex chromosome emergence

Of the 42 loci that demonstrate a sex-patterned profile in both data sets, 21 loci had the X- and Y-alleles conserved, while 13 loci had X- and Y-alleles switched. The 21 conserved X- and Y-alleles indicate that this sex chromosome had a shared ancestry between *O. niloticus* and *S. melanotheron*. The 13 loci where the X- and Y-alleles were switched, indicate that this period of shared ancestry was limited, and that some sites that underwent alternative lineage sorting as the proto-X and proto-Y were diverging. This indicates that speciation occurred soon after sex chromosome divergence.

### Evidence for structural rearrangements and their timing

The pronounced boundaries of divergence, high densities of sex-patterned SNPs and predicted deleterious mutations above the background level in *O. niloticus*, when taken together with the knowledge that chromosomes of this species are homomorphic in cytogenetic analyses, provide strong evidence for an inversion between the X- and Y-chromosomes in *O. niloticus* [33]. The absence of similar sharp boundaries in *S. melanotheron*, along with lower densities of sex-patterned SNPs and predicted deleterious mutations, suggest that this inversion is likely not present in the *S. melanotheron* lineage. We suggest the most parsimonious scenario is that this inversion arose following the divergence of *O. niloticus* and *S. melanotheron*.

The differentiation between males and females on LG22 of *S. melanotheron* suggests an association between this linkage group and sex-determination. While the levels of differentiation are lower than those witnessed on LG1 between 10.1 and 28 Mb, LG22 has a high density of sex-patterned deleterious mutations and the overall level of differentiation is significantly higher than the genomic background. Currently, this patterned is consistent with four hypotheses: 1) The signal could be the result of a reciprocal translocation of similar sized fragments between LG1 and LG22 after the divergence of *O. niloticus* and *S. melanotheron*. A previous karyotyping study has concluded that the chromosomes are homomorphic and that *S. melanotheron* have the same chromosome count as *O. niloticus*, but this study might have failed to detect such a translocation [17]. 2) LG22 is in linkage disequilibrium with the LG1 XY system. This seems unlikely because of the strong selection needed to overcome linkage disequilibrium between two independently assorting chromosomes. 3) A region on LG22 epistatically contributes to sex-determination within LG1 in this family. Multiple sex-determination systems have been observed in some cichlid species, however no sex-determination loci been previously reported from LG22 in cichlids [7, 8, 34]. 4) The signal could be an artifact of the sampling structure. This also seems unlikely as it would suppose that two copies of LG22 were segregating in the family and that males disproportionately received more of one copy and females received almost none of that copy by chance meiotic events. We suggest that a reciprocal translocation from LG22 to LG1 following the divergence of *S. melanotheron* and *O. niloticus* is the most likely scenario for explaining the observed patterned of sex-patterned differentiation on LG22. Future cytogenetic studies may provide data to test this hypothesis.

We summarize our findings by proposing a model for the evolution of the LG1 sex chromosome in tilapia (Fig. 4). The sex-determination mechanism resided on LG1 in the common ancestor of *O. niloticus* and *S. melanotheron*. Early stages of sex differentiation encompassed a region on LG1 from 10.1 to 18.9 Mb. The region from either 18.9 or 21.7 to 23.6 Mb was maintained in linkage disequilibrium with sex-determination locus by sexually antagonistic selection [32]. Due to a lack of shared SNPs between 18.9 and 21.7 Mb, we are unable to determine whether this region was originally not part of the diverging region and recombination was reduced only in the *S. melanotheron* lineage (Fig. 4) or this region was originally part of a block of divergence and recombination was restored to it in the *O. niloticus* lineage. *Oreochromis niloticus* and *S. melanotheron* diverged shortly after the emergence of the novel sex-determination locus. We propose that in the *O. niloticus* lineage, the region from 10.1 to 18.9 Mb experienced a chromosomal inversion and began to rapidly accumulate mutations. In the *S. melanotheron* lineage, the region did not experience this inversion and the region of sex differentiation expanded to encompass a region from 10.1 to 28 Mb. In *S. melanotheron*, LG1 may have further experienced a translocation of a region from LG22.

### Candidate sex-determination mutations

We identified 21 shared SNPs with consistent sex-patterned profiles in *S. melanotheron* and *O. niloticus*, which represent candidate sex-determination mutations for this lineage. Two of these are located near previously identified sex-determination candidate genes, so we evaluated how these shared sex-patterned SNPs might alter transcription factor binding sites. The mutation near *Ras association domain containing protein 10* did not affect any transcription factor binding sites known to play a role in sex-determination. The mutation near *Wilms tumor protein* altered a *Gata4* transcription factor binding site. *Gata4* is a transcription factor that has been associated with the sex-determination pathway [35]. *Gata4* has been shown to bind in the promoter of the *Wilms tumor protein* in mammals and teleosts and *Wilms tumor protein* is a strong activator of *Amhr*, a known sex-determination gene [36, 37]. A Y-specific loss of a *Gata4* binding site would decrease transcription of *Wilms tumor protein* in males, which in turn would not activate *Amhr* and result in masculinization. This variant currently represents the strongest SNP candidate for sex-determination on LG1 and further functional analysis is necessary to assess its role in sex-determination.

Our analysis is limited by the inability of the short-read Illumina data to clearly resolve the large number of deletions and insertions that are likely present on the Y-chromosome. Future studies should consider using longer read sequencing technologies to assemble X- and Y-specific sequences for both species. It may then be possible to more clearly identify conserved insertion and deletion events on the proto-Y chromosome in both species.

## Conclusions

This study utilized a comparative approach in order to identify the ancestral state of the LG1 sex-determination locus. We determined that *S. melanotheron* shares an ancestral XY sex-determination mechanism with *O. niloticus*. In both species, the highest differentiation between the X- and Y-chromosomes is found in the region from 10.1 to 18.9 Mb on LG1. We identified 21 sex-patterned SNPs shared between the two species. One of these SNPs alters a *Gata4* transcription factor binding site near *Wilms tumor protein*, which might alter the function of the sex-determination pathway. Future studies should assess the role of this candidate SNP in sex-determination. Investigation of additional species segregating this sex-determination system on LG1 will provide a clearer understanding of evolutionary processes during the early stages of sex chromosome divergence.

## Additional files

Additional file 1: Stop codon altering sex-patterned SNPs in *S. melanotheron*. (XLSX 39 kb)
Additional file 2: Stop codon altering sex-patterned SNPs in *O. niloticus*. (XLSX 37 kb)
Additional file 3: Shared sex-patterned SNPs between *O. niloticus* and *S. melanotheron* within intergenic region and the genes on either side of each shared sex-patterned SNP. *These shared sex-patterned SNPs had a gene on one side and the end of the scaffold on the other side (XLSX 32 kb)
Additional file 4: Shared sex-patterned SNPs between *O. niloticus* and *S. melanotheron* within intragenic regions and their position within those genes. (XLSX 47 kb)
Additional file 5: JASPAR analysis of transcription factor binding sites in the regions encompassing the shared SNPs. The uppercase letters in the sequence columns represent the SNP in question. The bolded SNPs are the SNPs within 20 kb of previously identified candidate genes for sex-determination. (XLSX 81 kb)

## Abbreviation

LG: Linkage group
